# Agreement of offspring-reported parental smoking status: the RHINESSA generation study

**DOI:** 10.1186/s12889-019-6414-0

**Published:** 2019-01-21

**Authors:** Kathrine Pape, Cecilie Svanes, Andrei Malinovschi, Bryndis Benediktsdottir, Caroline Lodge, Christer Janson, Jesus Moratalla, José Luis Sánchez-Ramos, Lennart Bråbäck, Mathias Holm, Rain Jögi, Randi Jacobsen Bertelsen, Torben Sigsgaard, Ane Johannessen, Vivi Schlünssen

**Affiliations:** 1 Section for Environment, Occupation and Health, Department of Public Health, Aarhus University, Aarhus, Denmark; 2 National Research Center for the Working Environment, Lersø Parkallé 105, DK-2100 Copenhagen O, Denmark; 3 Department of Medical Sciences: Clinical Physiology, Uppsala University, Uppsala, Sweden; 4 University of Iceland, Medical Faculty, Iceland. Primary Health Care Center, Gardabaer, Iceland; 5 Centre for Molecular, Environmental, Genetic & Analytic (MEGA) Epidemiology, University of Melbourne, Melbourne, Australia; 6 Center for International Health, Department of Global Public Health and Primary Care, University of Bergen, Bergen, Norway; 7 Department of Medical Sciences: Respiratory, Allergy and Sleep Research, Uppsala University, Uppsala, Sweden; 8 Department of Internal Medicine, Albacete University Hospital, Albacete, Spain; 9 Department of Nursing, University of Huelva, Huelva, Spain; 10 Department of Public Health and Clinical Medicine, Umeå University, Umeå, Sweden; 11 Occupational and Environmental Medicine, Sahlgrenska Academy, University of Gothenburg, Gothenburg, Sweden; 12 Department of Pulmonary Medicine, Tartu University, Tartu, Estonia; 13 Research Center Borstel, Leibniz-Center for Medicine and Biosciences, Division of Experimental Asthma Research, University of Kiel, Kiel, Germany; 14 Department of Occupational Medicine, Haukeland University Hospital, Bergen, Norway; 15 Department of Clinical Science, University of Bergen, Bergen, Norway

**Keywords:** Generation study, Validation study, Tobacco smoking, Self-report, Smoking during pregnancy, Parental smoking, Agreement, Sensitivity, Specificity

## Abstract

**Background:**

With increasing interest in exposure effects across generations, it is crucial to assess the validity of information given on behalf of others.

**Aims:**

To compare adult’s report of their parent’s smoking status against parent’s own report and examine predictors for discrepant answers.

**Methods:**

We studied 7185 offspring (18-51 years) and one of their parents, *n* = 5307 (27-67 years) participating in the Respiratory Health in Northern Europe, Spain and Australia (RHINESSA) generation study. Information about parent’s smoking status during offspring’s childhood and mother’s smoking status during pregnancy was obtained by questionnaires from parents and their offspring. We calculated sensitivity, specificity and Cohen’s Kappa [κ] for agreement using parent’s own report as the gold standard. We performed logistic regression to examine if offspring’s sex, age, educational level, asthma status, own smoking status or parental status, as well as the parent’s sex and amount of smoking during childhood predicted disagreement.

**Results:**

The sensitivity for offspring’s correct report of parent’s smoking status during childhood (0-10 years) was 0.82 (95% CI 0.81–0.84), specificity was 0.95 (95% CI 0.95–0.96) and a good agreement was observed, κ = 0.79 (95% CI 0.78–0.80). Offspring’s report of mothers’ smoking status during pregnancy showed a lower sensitivity, 0.66 (95% CI 0.60–0.71), a slightly lower specificity, 0.92 (95% CI 0.90–0.95) and a good agreement, κ = 0.61 (95% CI 0.55–0.67). In multivariate logistic regression analysis, offspring *not* having children was a predictor for discrepant answers (odds ratio [OR] 2.11 [95% CI 1.21–3.69]). Low amount of parents’ tobacco consumption, < 10 cigarettes/day (OR 2.72 [95% CI 1.71–4.31]) also predicted disagreement compared to ≥10 cigarettes per day, and so did offspring’s reports of fathers’ smoking status (OR 1.73 [95% CI 1.09–2.74]) compared to mothers’ smoking status. Offspring’s sex, asthma status, educational level, smoking status or age was not related to discrepant answers.

**Conclusions:**

Adults report their parent’s smoking status during their childhood, as well as their mother’ smoking status when pregnant with them, quite accurately. In the absence of parents’ direct report, offspring’s reports could be valuable.

**Electronic supplementary material:**

The online version of this article (10.1186/s12889-019-6414-0) contains supplementary material, which is available to authorized users.

## Background

Smoking is a major cause of respiratory diseases [1] and information on smoking habits is crucial in health research. Self-reported information on smoking is mostly used, despite the risk of underreporting of smoking, as measurements of biomarkers are often unavailable [2–4], and there is evidence suggesting that self-reported smoking exposure is quite accurate [4–6].

Increasing evidence suggests that exposures early in life influence subsequent health, and emerging evidence furthermore suggests that exposures even before conception, may influence the health of future generations [7, 8]. With increasing interest in exposure effects across generations, it is crucial to assess the validity of information given on behalf of others. In the absence of parents’ own report, offspring’s reports could be of major value in studies depending on exposures associated with previous generations.

A number of studies have examined offspring-reported parental information on history of somatic and mental diseases [9–11]; however, only one study [4], restricted to mother-daughter pairs from the Nurses’ Health Study II, has examined the accuracy of offspring-reported parental smoking status. The study found a high agreement of daughter’s reports of mother’s smoking status during pregnancy and childhood and the mother’s own report of smoking during pregnancy [4].

The objective of this population-based study is to examine the agreement of offspring-reported parental smoking status prenatally and during the offspring’s childhood, with mothers’ and fathers’ own self-reported smoking status. Furthermore, the study also aims to investigate predictors for discrepant answers in order to contribute to the field with knowledge of potential pitfalls in information given on behalf of others.

## Methods

### Study population

The study population is from the RHINESSA generation study - Respiratory Health in Northern Europe, Spain and Australia (www.rhinessa.net). One parent of each offspring in RHINESSA had previously participated in the Respiratory Health in Northern Europe (RHINE) study (www.rhine.nu), part of the European Community Respiratory Health Survey (ECRHS) initiated in 1989–1992 with three study waves (www.ecrhs.org) performed ten years apart. RHINE data was collected from seven different centres in five countries: Reykjavik (Iceland), Bergen (Norway), Umeå, Uppsala and Göteborg (Sweden), Aarhus (Denmark) and Tartu (Estonia). Furthermore, offspring with parents from three additional ECRHS centres, namely Melbourne (Australia), and Huelva and Albacete (Spain), were included in the RHINESSA population. Written consent was obtained from each participant in all study centres. In total, 8260 offspring from the RHINESSA cohort were matched with one of their parents (*n* = 6045). We used self-reported questionnaire information about smoking status from the parents themselves (collected in RHINE III/ECRHS III (2010–2012) or RHINE II/ECRHS II (1999–2000)) as well as offspring-reported parental smoking status (collected, among adult offspring (> 18 years), 2013–2016). Smoking during pregnancy was assessed from a specific women’s questionnaire in the RHINE III study. The questionnaires used have been developed in the framework of ECRHS, RHINE and RHINESSA and are used in several studies before. All questionnaires are available online from the study webpages listed above.

We used information from 7185 adult offspring (18-51 years) with information on one of their parents, *n* = 5307 (27-67 years), obtained in prioritised order from either RHINE III, ECRHS III, RHINE II or ECRHS II with full information as the eligibility criterion. In the centres of Melbourne, Huelva and Albacete, all smoking data were assessed by interview-administered questionnaires while data from all other centres were mostly assessed by a self-administrated questionnaire.

**Fig. 1:**
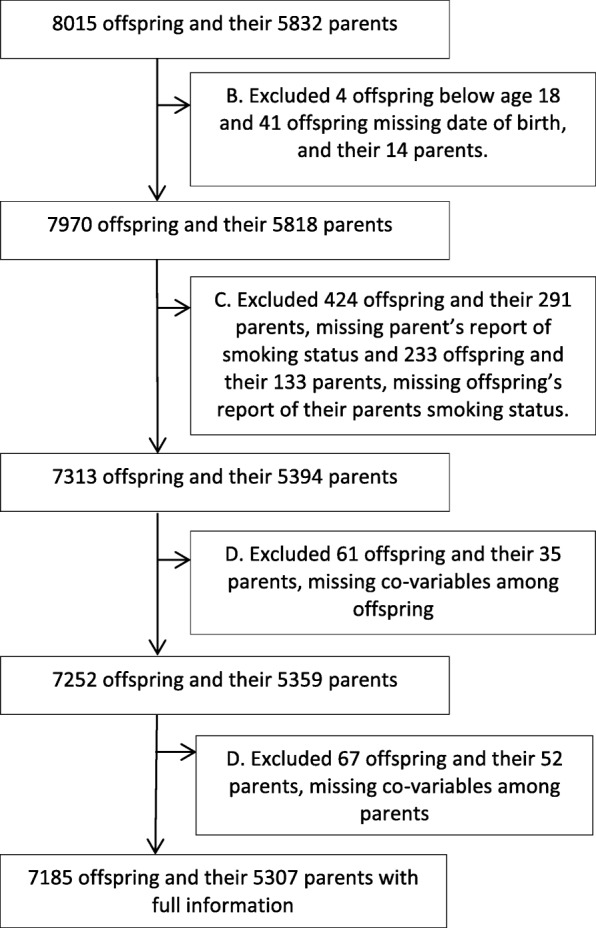
Flowchart of the exclusion process of the study population

A flowchart of the study population is provided in Fig. 1. Additionally, in the analysis of smoking during pregnancy, we used data from women participating in Rhine III, who also answered the questionnaire on women’s health, 807 offspring and their mothers (*n* = 679).

### Reporting of smoking status

Parental smoking status during offspring’s childhood was defined based on the parents’ date of birth, age of smoking debut or current age and number of years they had smoked (current smokers) or the year they had quit (ex-smokers). Offspring’s childhood was defined as the age period 0–10 years.

#### Parents’ own report of smoking status

Parents’ report of their own smoking status during their offspring’s childhood was accessed slightly differently, do to slightly different questions, in the different surveys and study waves. The questions used were:

A. RHINE III: “*Do you smoke? (this applies even if you only smoke the odd cigarette/cigar or pipe every week) (No/Yes)”, “Did you smoke previously? (No/Yes)”*, *“How old were you when you started smoking? (...years)”, “For how long have you smoked? (applies to both smokers and ex-smokers) (...years)”*, *and “If you are an ex-smoker, when did you stop smoking? Year ...”*
B. RHINE II: “*Are you a smoker (this applies even if you only smoke the odd cigarette/cigar or pipe every week)? (No/Yes)”*, *“Are you an ex-smoker? (No/Yes)”*, “*Smoked for …years (applies to both smokers and ex-smokers)”, and “Stopped smoking in … (year)”*
C. ECRHS III and ECRHS II: “*Have you ever smoked for as long as a year? [‘YES’ means at least 20 packs of cigarettes or 12 oz. (360 grams) of tobacco in a lifetime, or at least one cigarette per day or one cigar a week for one year] (No/Yes)”, “How old were you when you started smoking? (Years)”, “Do you now smoke, as one month ago? (No/Yes)”, “Have you stopped or cut down smoking? (No/Yes)”,* and *“How old were you when you stopped or cut down smoking? (Years)”*

#### Mothers’ own report of smoking status during pregnancy

In the “*RHINE III – Women’s questionnaire”,* mothers’ report of own smoking status during pregnancy was identified through the question: *“During this pregnancy (tick if yes)… Did you smoke?”* The mothers’ report of own smoking status during pregnancy was dichotomized according to whether or not they smoked, and correct pregnancy (in case of siblings) was identified through offspring’s birth year.

#### Offspring-reported parental smoking status

In the RHINESSA study, offspring-reported parental smoking status during the offspring’s childhood was obtained through the questions “*Did your father ever smoke regularly during your childhood? (No/Yes/Don’t know)”, “Did your mother ever smoke regularly during your childhood? (No/Yes/Don’t know)”.* Their mothers’ smoking status during pregnancy, on the other hand, was obtained through the question “*Did your mother smoke when she was pregnant with you? (No/Yes/Don’t know)”.* Offspring-reported parental smoking status during the offspring’s childhood and offspring-reported maternal smoking status during pregnancy was dichotomized as “Yes” or “No” and the “Don’t know” category was treated as missing data. For 2.3% of the fathers and 1.4% of the mothers, their offspring answered “Don’t know” to the question on whether they had smoked during the offspring’s childhood, while 23.1% of the mothers’ smoking status during pregnancy was categorized as “Don’t know” by the offspring.

### Predictors for disagreement

Offspring’s sex, age, educational level, asthma status (self-reported doctor diagnosed asthma), own smoking status and own parental status were included as potential predictors for disagreement. Offspring’s sex was included in the model to examine sex differences in reporting of their parent’s smoking; age was included to examine the differences in time trends and risk of recall bias as a predictor for discrepant answers. Educational level was included to examine if the well-studied “health-education gradient” would be a predictor for awareness of parents’ health behaviour. Offspring own asthma status was included as we hypothesise children’s own asthma status will influence their recall of their parents smoking. Offspring’s own smoking status and parental status were included to examine if their own behaviour were increasing their awareness about their parents’ behaviour. Asthma, smoking status and own parental status were dichotomized as “No” or “Yes”. Offspring’s age was categorized as “< 25 years”, “≥25 < 35 years” or “≥35 years” (reference). Education was categorised as “primary school” (reference), “secondary school/technical school”, or “college or university”. Parents’ sex and average amount of smoking were also included in the analyses as predictors for disagreement. Parents’ sex was included in the model to examine any sex differences, where amount of smoking was included to examine if higher amount of smoking had increased the awareness among the offspring. Parents’ amount of smoking was obtained in the surveys based on the following questions:

A. RHINE III: *“How much do you smoke / did you smoke? (give an average …cigarettes/day,…cigars/week, …pkts pipe tobacco/week”*
B. RHINE II: *“Smoke/smoked …cigarettes/week, …cigars/week, …pkts pipe tobacco/week”*
C. ECRHS III and II: *“On average* *of the entire time you smoked, before you stopped or cut down, how much did you smoke? Number of cigarettes per day…, number of cigarillos per day…, numbers of cigars a week…, pipe tobacco in b) grams/week…”.*

The different tobacco products were converted to cigarettes. One cigarillo was converted to three cigarettes, one cigar to five cigarettes, and one gram pipe tobacco equalled one cigarette [12]. The amount of smoking was dichotomized as 10 or more cigarettes per day or less than 10 cigarettes per day on average.

### Statistical procedures

Sensitivity and specificity were calculated with 95% confidence intervals [CIs]. Cohen’s Kappa estimate [K], with 95% CIs, was calculated to estimate the agreement of the offspring-reported parental smoking status during childhood and pregnancy with the parent reported smoking status during the same periods. Parents’ own report was considered the gold standard. Cohen’s Kappa interpretation was based on the following categories: poor agreement, < 0.2; fair, 0.21–0.40; moderate, 0.41–0.60; good, 0.61–0.80; and very good, 0.81–1.00 [13]

Multivariate logistic regression models were conducted to estimate the odds ratio [OR] of whether offspring’s sex, age, educational level, asthma status, own smoking status or own parental status and parents’ sex and amount of smoking were predictors for disagreement. The regression models were performed with repeated measurements due to multiple offspring from the same parent using proc. GENMOD function in SAS. The model was mutually adjusted for the included variables and further adjusted for study centre. Disagreement was defined as discrepant answers between offspring and their parents. The significance level was set at a *p-*value of < 0.05 (two-sided) and 95% CIs were calculated.

We performed sensitivity analyses where those excluded due to missing co-variables were included in the calculation of sensitivity, specificity and K. Furthermore, we performed sensitivity analyses where childhood was expanded to cover 0–18 years. We performed analysis by sex of the parents, and by the offspring’s own parental status. Subgroup analyses were conducted to examine if parents’ *amount* of smoking affected the sensitivity, specificity, or agreement. Further subgroup analyses were performed to examine whether false answers from the main analyses were false positives or false negatives. Additionally, sensitivity analysis with a reversed priority line (1. RHINE II, 2. ECRHS II, 3. RHINE III, and 4. ECRHS III) of the included data from the parents was performed to examine if the period of the data collection and perception of smoking at that time had an impact on the estimates. Separate analyses of centres were conducted for sensitivity, specificity and K, and adjustment for study centre was applied in the logistic regression models. All statistical analyses were performed using SAS (SAS Institute Inc., Cary, NC, USA) version 14.1.

## Results

Baseline characteristics of the 7185 offspring and 5307 parents are presented in Table 1. Offspring had a mean age of 30 years when they answered the RHINESSA questionnaire. The parents’ mean age was 53 years. Slightly more women than men participated, both among offspring (58%) and parents (55%). Most of the offspring had a higher education (60%), 12% were smokers, 16% had asthma and 41% had children themselves. Forty-one percent of parents smoked during the offspring’s childhood, of those 54% had on average smoked at least 10 cigarettes per day.

**Table 1:** Characteristics of offspring and parents

Offspring, *n* = 7185		
Female, *n (%)*	4147	(57.7)
Age, *mean (SD)*	30	(7.7)
Smoker, *n (%)*	877	(12.2)
Asthma, *n (%)*	1157	(16.1)
University/College, *n (%)*	4281	(59.6)
Secondary, *n (%)*	2693	(37.5)
Primary, *n (%)*	211	(2.9)
At least one child, *n (%)*	2920	(41.1)
Parents, *n* = 5307		
Female, *n (%)*	2931	(55.2)
Age, *mean (SD)*	53	(7.2)
Smoking during offspring’s childhood, *n (%)*	2195	(41.4)
> 10 cig, *n (%)*	1191	(53.5)
< 10 cig, *n (%)*	1035	(46.5)

The sensitivity for correct offspring-reported parental smoking status during childhood (0-10years) was 0.82 (95 % CI 0.81–0.84), specificity was 0.95 (95 % CI 0.95–0.96) and a good agreement was observed κ = 0.79 (95 % CI 0.78–0.81) (Table 2). Offspring’s report of mothers’ smoking status during pregnancy showed a lower sensitivity 0.66 (95 % CI 0.60–0.71), and a slightly lower specificity 0.92 (95 % CI 0.90–0.95) compared to the analysis during childhood, and a good agreement κ = 0.61 (95 % CI 0.55–0.67) (Table 2).

**Table 2:** Sensitivity, specificity and Cohen’s Kappa estimate of smoking status prenatal or during offspring’s childhood

Parents’ smoking status during offspring’s childhood 0–10 years (offspring *n* = 7185, parents *n* = 5307)		
		95% CI
Sensitivity	0.82	[0.81;0.84]
Specificity	0.95	[0.95;0.96]
Cohen’s Kappa	0.79	[0.78;0.80]
Mothers’ smoking status during pregnancy (offspring *n* = 807, mother n = 679)		
		95% CI
Sensitivity	0.66	[0.60;0.71]
Specificity	0.92	[0.90;0.95]
Cohen’s Kappa	0.61	[0.55;0.67]

*CI* confidence interval

Multivariate logistic regression analyses (Table 3) showed that offspring’s own status as a parent was a predictor for discrepant answers, where offspring with no children had a higher disagreement (OR 2.11 [95% CI 1.21–3.69]) compared to offspring with children. Younger offspring age tended to predict discrepant answers (OR 2.03 [95% CI 0.95–4.35]) for offspring < 25 years. A lower amount of smoking was related to more discrepant answers, < 10 cigarettes per day (OR 2.72 [95% CI 1.71–4.31]) compared to 10 or more cigarettes per day. Offspring’s report of their fathers’ smoking status was also found to be a predictor for discrepant answers (OR 1.73 [95% CI 1.09–2.74]) compared to offspring’s report of their mothers’ smoking status during offspring’s childhood. Offspring’s own sex, asthma status, educational level or smoking status was not significantly related to discrepant answers.

**Table 3:** Predictors for discrepant answers for offspring- reported parental smoking status and parents own report of smoking

Predictors for disagreement		
	OR	95% CI
Offspring sex		
Female	1.00	
Male	1.06	[0.66;1.70]
Offspring asthma status		
Asthma	1.00	
No asthma	1.04	[0.57;1.92]
Offspring educational level		
Primary	1.00	
Secondary	4.32	[0.64;29.20]
University/College	5.06	[0.69;34.45]
Offspring smoker status		
No	1.00	
Yes	1.42	[0.69;2.91]
Offspring age		
≥ 35 years	1.00	
≥ 25 < 35 years	1.30	[0.72;2.35]
< 25 years	2.03	[0.95;4.35]
Offspring’s own parental status		
Children	1.00	
No children	2.11	[1.21;3.67]
Parents sex		
Mother	1.00	
Father	1.73	[1.09;2.74]
Parents amount of smoking		
≥ 10 cig /day	1.00	
< 10 cig /day	2.72	[1.71;4.31]

Analysis performed with multivariate logistic regression analysis. *CI* confidence interval, *OR* Odds ratio, variables mutually adjusted and adjusted for study centre

### Sensitivity analyses

Sensitivity analyses including those who were excluded due to missing covariates produce similar results, sensitivity 0.82 (95% CI 0.81–0.84), specificity 0.95 (95% CI 0.95–0.96) and κ 0.79, (95% CI 0.77–0.80). When childhood was expanded to cover 0–18 years, similar results were also observed, sensitivity 0.82 (95% CI 0.80–0.83), specificity 0.96 (95% CI 0.95–0.96) and κ 0.79, (95% CI 0.77–0.80).

Analyses by sex showed largely similar results with a slightly lower sensitivity 0.81 (95% CI 0.79–0.83) and κ 0.78, (95% CI 0.76–0.80) and similarly specificity 0.96 (95% CI 0.95–0.97) of the fathers compared to mothers, sensitivity 0.84 (95% CI 0.82–0.86), κ 0.80, (95% CI 0.78–0.82) and specificity 0.95 (95% CI 0.94–0.96), Additional file 1: Table S1. Analyses by offspring’s own parental status showed a lower sensitivity 0.78 (95% CI 0.76–0.80), and κ 0.77, (95% CI 0.75–0.79) and similar specificity 0.96 (95% CI 0.96–0.97) of the offspring without children, compared to offspring with children, sensitivity 0.87 (95% CI 0.85–0.88), κ 0.80, (95% CI 0.77–0.82) and specificity 0.93 (95% CI 0.92–0.94).

In subgroup analyses of parents’ smoking amount, the sensitivity 0.89 (95% CI 0.88–0.91) and κ 0.85, (95% CI 0.83–0.86) were increased when the parents had smoked more than 10 cigarettes per day during their offspring’s childhood compared to the main analysis and decreased if the parents had smoked less than 10 cigarettes per day, sensitivity 0.74 (95% CI 0.72–0.76) and κ 0.72, (95% CI 0.70–0.74). The specificities remained unchanged. Separate analysis of offspring and parental predictors for disagreement produced similar results as the main analyses with offspring and parental predictors in the same model.

Further analyses of the 715 false answers in the main analysis showed a higher prevalence of false negative answers (*n* = 511, 71%) compared to false positive answers (*n* = 204, 29%), meaning that the parents had reported smoking more often than the offspring. Characteristics of offspring themselves who reported false negative answers were similar to the total study population except higher educational level (University/College 66% vs 60%), whereas offspring who reported false positive answers were slightly older and more often females (59% vs 58%), smokers (17% vs 12%), and asthmatics (18% vs 16%). Offspring who reported false negative were less likely to report regarding their mothers (49% vs 55% females) compared to the total population, whereas offspring who reported false positive answers were more likely to report regarding their mothers (60% vs 55% females). Parents’ mean age was slightly higher for offspring who reported false negative as well as false positive compared to the total population (54 and 55 vs 53 years).

Sensitivity analysis of the reversed data priority produced similar sensitivity, specificity and Cohen’s Kappa as for the main analysis.

Analyses stratified by study centre with respect to sensitivity, specificity and Cohen’s Kappa showed largely the same result as for the combined analysis; however, with wider confidence intervals with kappa ranging from 0.71 to 0.83 (Additional file 2: Table S2).

## Discussion

Results from this generation-based cohort study suggest that offspring quite correctly report their parents’ smoking status compared to parents’ own report. Offspring-reported parental smoking during childhood demonstrates a good agreement as well as for reports of mothers’ smoking status during pregnancy. Recall of childhood exposures may be a reliable source of exposure information for studies investigating generational exposures, where parental reports are not available.

Predictors for discrepant answers in our study were offspring’s own parental status, parents’ amount of tobacco consumption and sex of the parents, whereas offspring’s own sex, age, educational level, asthma status, or smoking status did not predict discrepant answers.

A reasonable explanation for different levels of agreements between offspring’s report of parents’ smoking status during childhood (K = 0.79) compared to reports of their mothers’ smoking status during pregnancy (K = 0.61) could be that the offspring have more direct information available during their own childhood compared to during pregnancy where the offspring solely have the information from a second source; e.g. the mother herself or other family members. Of the offspring 23% reported “Don’t know” to the question of whether their mother smoked during pregnancy or not, compared to 2% of reports during childhood which also indicates a higher uncertainty for mothers’ smoking status during pregnancy. Another possible explanation is the mother’s shame that she was smoking during pregnancy and therefore incorrectly reported that she did not smoke during pregnancy. Still, offspring’s report of the mother’s smoking status during pregnancy is documented to be a useful proxy for the mother’s own report of smoking during pregnancy.

To our knowledge, this is the first study to examine the agreement of offspring-reported parental smoking status in a general population of both mothers and fathers. The sensitivity, specificity and agreement for the mothers’ smoking status during pregnancy are roughly in line with findings of an earlier study [4] of daughter’s reports of mother’s smoking during pregnancy compared to the mother’s own report. They found a sensitivity ranging from 74 to 85%, specificity 90 to 95% and κ between 0.72 and 0.81. The study was based on mother-daughter pairs participating in the Nurses’ Health Study II in contrast to our population-based cohort. The higher agreement in the Nurses’ Health Study could reflect the participants’ occupation as nurses, which could make them more aware of health, e.g. smoking habits, than participants from a general population.

### Sex of the parents

The offspring were more likely to give a correct answer regarding their mothers’ than their fathers’ smoking status, which may be explained by the fact that mothers in general spend more time with their children, especially in early life, and consequently the offspring are more aware of their mothers’ smoking status. A similar argument was used by the “National Heart, Lung, and Blood Institute - Family Heart Study” of offspring-reported parental history of chronic heart diseases and diabetes that demonstrated high agreement with parental self-reported history; however, a lower agreement for asthma and hypertension [10], where they suggested that conditions which affect “daily routines such as medication use or diet changes” also give relatives more contact and awareness of the diseases. However, gender roles have changed over the years, especially in Northern Europe, and today, it is not always true that women spend more time than men at home with their children. However, data in the present study are based on offspring born between 1963 and 1998, and women are today still more likely to have custody of their children after a divorce [14], which could be part of the explanation of why the offspring are more aware of the mothers’ smoking status.

### Amount of parental tobacco consumption

A low amount of parental tobacco consumption predicted discrepant answers confirming the results of Simard et al. [4]. The more the parents smoked, the more likely it is that children noticed their parents’ smoking habits. A limitation of the questions on smoking amount is that it is not specific for the offspring’s childhood, but reported as a long-term average, which may have varied over the lifetime of the parent’s smoking which could introduce a potential for misclassification.

We found a quite high sensitivity and an even higher specificity indicating a low risk of misclassifying non-smokers, while the risk of misclassifying smokers is higher but still limited. In line with these findings, we could see a higher prevalence of false negative answers compared to false positive answers among the offspring which may be due to the increasing awareness of smoking and second-hand smoking’s negative influence on health [15], and parents’ influence on children’s later smoking behaviour [16]. The use of indirect information could, to a higher extent, introduce misclassification of smokers compared to non-smokers and therefore underestimate the true prevalence of smokers.

### Offspring

Offspring’s own parental status was shown to be a predictor for discrepant answers. A higher sensitivity and agreement was found among offspring with children themselves. These results could indicate that offspring with children are more aware of exposures in the past, and may start to ask their parents about their own childhood. Even though younger participants had a shorter recall period, the younger the offspring, the higher the odds ratio of discrepant answers, however, this was not significant. This could possibly be explained by the fact that perception of smoking has changed over time in society, e.g. greater knowledge about the harmful impacts of smoking on health [15] and parents could have avoided smoking in the vicinity of their children to a greater extent for the younger offspring. We chose to prioritize the use of the most recent data collected from parents so that answers from both parents and children reflected similar societal attitudes with respect to smoking. The sensitivity analysis of a reversed priority in preferred data provided similar results.

### Limitations and strengths

Using parents’ own self-reported information as the gold standard probably introduces a risk of underestimation of the true prevalence of smoking [2–4]. The numbers of parents who smoked during the offspring’s childhood and pregnancy were based on participants from numerous Western countries as well as Australia and from several different decades which makes it difficult to compare the percentages of smokers with the known prevalence of specific countries or time periods.

In our study, the analysis of the discrepant answers between offspring and their parents was primarily false negative answers. The underestimation of the offspring’s report in addition with the lack of a true gold standard (e.g. saliva/urinary cotinine) will enhance the risk of underestimating the true prevalence of smoking. A consequence is that smoking information given on behalf of others is not ideal if the purpose of a study is to determine prevalence of smoking, but it can be useful in investigations researching associations between smoking and diseases, even though some misclassification can be expected.

A limitation of the study is that the wording for parents own report of smoking (which picks up a measure of “current” use) is different to the wording for the offspring-reported parental smoking (which picks up a measure of “regular” use), which is not identical concepts. For example, a parent who smoked very intermittently may consider themselves a smoker, but the offspring may not see this as regular use.

We do not have information on whether the offspring who completed self-administrated questionnaires consulted their parents to provide answers about parental smoking. However, we are reassured by finding no clear evidence of difference from centre specific analysis. The measures of agreement from centres based solely on interview data (where parental consultation was not possible) (Melbourne, Huelva, and Albacete) did not substantially differ from centres that collected data from self-administrated questionnaires, Additional file 2: Table S2.

A major strength of our study is the large population-based RHINESSA population, which enabled us to examine both male and female offspring as well as both male and female parents from a general population. A further strength is the ability to determine predictors for discrepant answers based on the large amount of available information from both parents and offspring.

## Conclusion

This study demonstrates that adult offspring with good agreement reported their parents’ smoking status during their own childhood and their mothers’ smoking status during pregnancy compared to their parents’ own answer. Offspring’s own parental status, the amount of parental smoking as well as sex of the parents predicted disagreement while offspring’s own sex, age, educational level, asthma status, or smoking status did not influence the risk of discrepant answers. Our results suggest that in the absence of smoking information from parents themselves, offspring’s reports could be valuable.

## Additional files

Additional file 1: **Table S1.** Sensitivity, specificity and Cohen’s Kappa estimate of smoking status during offspring’s childhood by sex. (DOCX 14 kb)

Additional file 2: **Table S2.** Sensitivity, specificity and Cohen’s Kappa estimate of smoking status during offspring’s childhood by centre (DOCX 15 kb)

## Abbreviations

CI: Confidence interval
ECRHS: European Community Respiratory Health Survey
K: Cohen’s Kappa
OR: Odds ratio
RHINE: Respiratory Health in Northern Europe
RHINESSA: Respiratory Health in Northern Europe, Spain and Australia
