# Supplementary material for: Agreement of offspring-reported parental smoking status: the RHINESSA generation study
Source: BMC Public Health. 2019 Jan 21;19:94. doi: 10.1186/s12889-019-6414-0 (PMC6341700; doi:10.1186/s12889-019-6414-0)
Supplement: Supplementary file 1 — Table S1. Sensitivity, specificity and Cohen’s Kappa estimate of smoking status during offspring’s childhood by sex. (DOCX 14 kb) [file 12889_2019_6414_MOESM1_ESM.docx]

**Table S1** Sensitivity, specificity and Cohen’s Kappa estimate of smoking status during offspring’s childhood by sex.

| **Fathers’ smoking status during offspring's childhood 0-10 years (offspring *n* = 7185, parents *n* = 5307)** | | | | | | |
| --- | --- | --- | --- | --- | --- | --- |
| **95% CI** | | | | | | |
| Sensitivity | 0.81 | [0.79;0.83] |  |  |  |  |
|  | Specificity | 0.96 | [0.95;0.97] |  |  |  |
|  | Cohen’s Kappa | 0.78 | [0.76;0.80] |  |  |  |
|  |  |  |  |  |  |  |
| **Mothers’ smoking status during offspring's childhood 0-10 years (offspring *n* = 807, mother n = 679)** | | | | | | |
|  | Sensitivity | 0.84 | **95% CI**  [0.82;0.86] |  |  |  |
|  | Specificity | 0.95 | [0.94;0.96] |  |  |  |
|  | Cohen’s Kappa | 0.80 | [0.78;0.82] |  |  |  |

CI, confidence interval.
