# Supplementary material for: Agreement of offspring-reported parental smoking status: the RHINESSA generation study
Source: BMC Public Health. 2019 Jan 21;19:94. doi: 10.1186/s12889-019-6414-0 (PMC6341700; doi:10.1186/s12889-019-6414-0)
Supplement: Supplementary file 2 — Table S2. Sensitivity, specificity and Cohen’s Kappa estimate of smoking status during offspring’s childhood by centre (DOCX 15 kb) [file 12889_2019_6414_MOESM2_ESM.docx]

Table S2 Sensitivity, specificity and Cohen’s Kappa estimate of smoking

status during offspring’s childhood by centre

| **Parents' smoking during offspring's childhood 0-10 years** | | | | | | | |
| --- | --- | --- | --- | --- | --- | --- | --- |
|  |  |  | Sensitivity [95% CI] |  | Specificity [95% CI] |  | Cohen’s Kappa [95% CI] |
| *Agreement* | |  | 0,82 [0,81;0,84] |  | 0,95 [0,95;0,96,] |  | 0,79 [0,78;0,80] |
|  | Albacete |  | 0,75 [0,62;0,88] |  | - |  | - |
|  | Bergen |  | 0,82 [0,79;0,85] |  | 0,96 [0,95;0,97] |  | 0,79 [0,75;0,82] |
|  | Goteborg |  | 0,84 [0,80;0,88] |  | 0,96 [0,77;0,85] |  | 0,81 [0,77;0,85] |
|  | Huelva |  | 0,91 [0,79;1,03] |  | 1,00 [1,00;1,00] |  | 0,83 [0,60;1,00] |
|  | Melbourne |  | 0,68 [0,51;0,84] |  | 1,00 [1,00;1,00] |  | 0,71 [0,55;0,87] |
|  | Reykjavik |  | 0,86 [0,83;0,89] |  | 0,92 [0,89;0,94] |  | 0,78 [0,74;0,82] |
|  | Tartu |  | 0,78 [0,72;0,84] |  | 0,93 [0,90;0,96] |  | 0,72 [0,66;0,79] |
|  | Umea |  | 0,85 [0,81;0,88] |  | 0,97 [0,95;0,98 |  | 0,83 [0,80;0,86] |
|  | Uppsala |  | 0,78 [0,74;0,82] |  | 0,97 [0,96;0,98] |  | 0,78 [0,74;0,82] |
|  | Aarhus |  | 0,83 [0,79;0,87] |  | 0,93 [0,90;0,95] |  | 0,76 [0,72;0,81] |

CI, confidence interval; Sensitivity and Cohen’s Kappa of Albacete is not calculated due to *n* < 3.
